# The tomato floral homeotic protein FBP1-like gene, *SlGLO1*, plays key roles in petal and stamen development

**DOI:** 10.1038/srep20454

**Published:** 2016-02-04

**Authors:** Xuhu Guo, Zongli Hu, Wencheng Yin, Xiaohui Yu, Zhiguo Zhu, Jianling Zhang, Guoping Chen

**Affiliations:** 1 Key Laboratory of Biorheological Science and Technology (Chongqing University), Ministry of Education, Bioengineering College, Chongqing University, Chongqing 400044, People’s Republic of China

## Abstract

MADS-box transcription factors play important role in plant growth and development, especially floral organ identities. In our study, a MADS-box gene *SlGLO1*- tomato floral homeotic protein FBP1-like gene was isolated. Its tissue-specific expression profile analysis showed that *SlGLO1* was highly expressed in petals and stamens. RNAi (RNA interference) repression of *SlGLO1* resulted in floral organ abnormal phenotypes, including green petals with shorter size, and aberrant carpelloid stamens. *SlGLO1*-silenced lines are male sterile. Total chlorophyll content was increased and chlorophyll biosynthetic genes were significantly up-regulated in *SlGLO1*-silenced petals and stamens. Furthermore, B-class genes expression analysis indicated that the repressed function of *SlGLO1* led to the enhanced expression of *TAP3* and the down-regulation of *TPI* in the petals and stamens, while the expression of *TM6* was reduced in petals and increased in stamens and carpels of *SlGLO1*-RNAi plants. Additionally, pollen grains of transgenic lines were aberrant and failed to germinate and tomato pollen-specific genes were down-regulated by more than 90% in *SlGLO1*-silenced lines. These results suggest that *SlGLO1* plays important role in regulating plant floral organ and pollen development in tomato.

ABC model of flower development was broadly accepted based on extensive genetic and molecular studies in several model plants^1^. A-class genes in the first floral whorl specify sepal identity. A- and B-class genes together are required for petal development, B- and C-class genes are both needed for stamen development, and C-class genes in the fourth whorl specify carpel identity. Additional regulatory functions have been found, such as D-class genes that are necessary for ovule identity^2^ and E-class genes that are required for proper floral organ identity in the different whorls^3^. In *Arabidopsis*, there are two A-class genes *APETALA1* (*AP1*) and *APETALA2* (*AP2*), two B-class genes *APETALA3* (*AP3*) and *PISTILLATA*) (*PI*), and one C-class gene *AGAMOUS* (*AG*). The E-class genes contain *SEPALLATA1* (*SEP1*), *2*, *3*, and *4*^3^. All of these genes are MADS box genes except for *AP2* and its homologues^4^. In tomato, *MACROCALYX* (*MC*) represents the A-class genes are involved in the development of sepals in the first whorl and in inflorescence determinacy^5^. Recent studies have suggested that the overexpression of *SlFYFL* represents longer sepals^6^. B-class MADS box genes specify petal and stamen identities in several core eudicot species, such as, *Solanum lycopersicum GLOBOSA* (*SlGLO*^7^) (syn. *SlGLO1*, *LePI*, *TPIB*^8^,^9^), *Tomato PISTILLATA* (*TPI*^10^) (syn. *SlGLO2*^7^), *Tomato MADS box gene 6* (*TM6*) (syn. *TDR6*^11^,^12^) and *Tomato APETALA3* (*TAP3*) gene (syn. *STAMENLESS*, *SlDEF*, *LeAP3*^13^,^10^,^14^). Both *TPI*- and *SlGLO1*-silenced plants exhibit aberrant carpelloid stamens while petals are unaffected^9^. A mutation in *TAP3* and the silencing of *TM6* both results in the conversion of stamens into carpels and a more or less severe conversion of petals into sepals^10^. Tomato C-class gene *TOMATO AGAMOUS 1* (*TAG1*) which specified stamen and carpel identities has been identified^15^. *Tomato MADS box gene 5* (*TM5*^16^) and *Tomato AGAMOUS-LIKE gene 2* (*TAGL2*) (syn. *TM29*^11^,^17^), the two E-class genes, have been described on the basis of their expression patterns and down-regulated phenotypes in tomato.

In tomato, several mutants also exhibit partial or complete homeotic transformations in the second and third floral organ whorls. The *sl-2*^18^, *sl*^19^, and *green pistillate* (*gpi*) (syn. *pi-2*, *pistillate 2*^20^) are the most investigated mutants for which petal and stamen identity were affected. The petals appear as normal in the *sl-2* mutant, while the stamens are twisted and distorted, bearing naked ovules. The *sl* mutant exhibits sepaloid petals and stamens being replaced by carpels in the third whorl^19^. The *gpi* mutant shows a strong and full homeotic transformation of petals into sepals and of stamens into carpels^20^. The study of Quinet *et al.*^13^ indicated that petal and stamen identity in tomato depends on gene-hormone interactions, as mediated by the *TAP3* gene through identifying the mutations responsible for the *sl* phenotypes and investigating how *TAP3* interacts with floral meristem identity genes and hormones in tomato.

Although function of many MADS box genes appear to be conserved among flowering plants, some homologous genes in different species have recruited novel functions through evolutionary mechanisms^21^. One key driving force in evolution is gene duplication^22^. Duplication of genes often provides a basis for diversification in either molecules or morphology. The diversification usually accompanies the definite fate of duplicated genes that either acquire a new role or undergo subfunctionalization. The Solanaceae possess multiple copies of *DEF* lineage and *GLO* lineage genes, allowing for diversification of function. The two duplicates for each lineage of B-class MADS-box genes were found to redundantly partition the role of the B function with a variable subfunctionalization process in *Petunia*, *Nicotiana* and *Solanum*^9^,^10^,^23^,^24^,^25^. Recently, specific *PFGLO2* silencing in *Physalis floridana* show no homeotic variation but rather affect pollen maturation, providing insights into functional divergence of the *GLOBOSA* duplicates within the Solanaceae^26^. The B-class MADS box genes *GLO* and *DEF* together control the organogenesis of petals and stamens. Quantitative levels of B-class genes expression required to obtain petal tissue related to single versus duplicated genes. In plants where the B function genes are duplicated, there is higher redundancy but still quantitative changes in gene expression beyond a certain level also cause observable phenotypes. Changes in gene expression show expression thresholds required for organ formation^27^,^28^. In model plant *Antirrhinum*, thresholds of 11–15% of wild-type levels of expression of *DEF* or *GLO* are associated with development of recognizable petal tissue^28^, supporting the importance of quantitative gene expression levels for floral patterning and organ development.

Previous study by Geuten and Irish showed tomato *SlGLO1*-RNAi was weakly affected in stamen development in tomato cv. Micro-Tom^9^. Because Micro-Tom plants has three mutations at least in *self-pruning* (*sp*), *dwarf* (*d*) and *miniature* (putative *mnt*) genes^29^,^30^ based on its pedigree and the characterization of one of them, *putative mnt*, has still been not elucidated, the biochemical and molecular function of *SlGLO1* still need further elucidation in tomato cv. Ailsa Craig (a common material for studying tomato). In our study, based on different genetic background we used tomato cv. Ailsa Craig, as the research material which is normal height compared with Micro-Tom. To comprehensively examine the diversification of functions of B-class genes in the Solanaceae, We have isolated *SlGLO1* gene (a tomato floral homeotic protein FBP1-like gene) from wild-type tomato (*Solanum lycopersicon* Mill. cv. Ailsa Craig) flowers and examined its tissue-specific expression. An RNA interference (RNAi) expression vector targeting *SlGLO1* was constructed and transformed into tomato plants. Silencing of *SlGLO1* in tomato leads to floral organ abnormal phenotypes and defective pollen development, Furthermore, we analyzed the silencing lines at both physiology and molecular levels. This article enhanced our knowledge about the roles of *SlGLO1* playing in diverse developmental processes.

## Results

### *SlGLO1* isolation and expression pattern analysis

**Figure 1:**
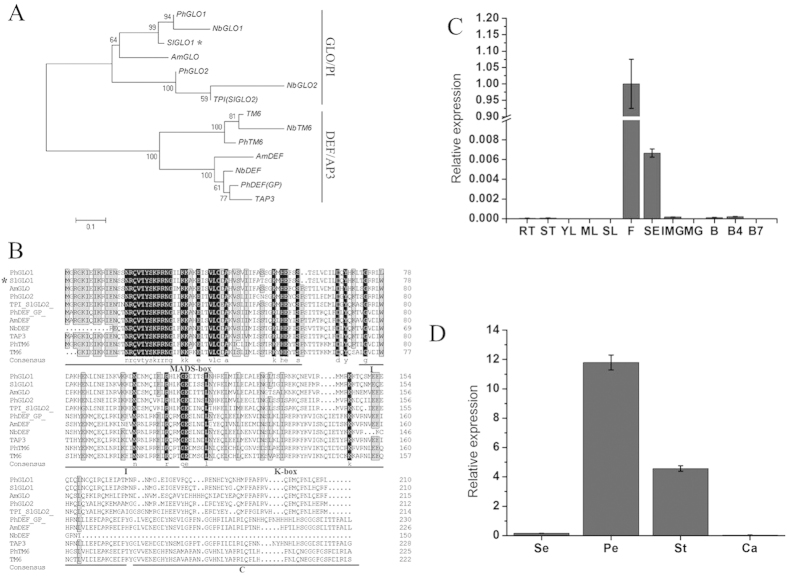
Sequence and expression analysis of *SlGLO1* in WT. (**A**) Phylogenetic analysis of *SlGLO1* and other MADS-Box proteins was performed by the neighbor-joining method, bootstrap analysis of 1,000 replicates. SlGLO1 is marked with asterisk. Accession numbers for other proteins are listed as follows: PhGLO1 (Q03488.1), NbGLO1 (HQ005417), AmGLO (Q03378.1), PhGLO2 (CAA49568.1), NbGLO2 (HQ005418), TPI (DQ674531), TM6 (X60759), NbTM6 (AY577817), PhTM6 (AAS46017.1), AmDEF (CAA44629.1), NbDEF (DQ437635), PhDEF (Q07472), TAP3 (DQ674532). (**B**) Multiple sequence alignment of SlGLO1 and other MADS-Box proteins. SlGLO1 is marked with asterisk. Identical amino acids are shaded in black, and similar amino acids are shaded in gray. (**C**) The relative expression patterns of *SlGLO1* in WT. RT, root; ST, stem; YL, young leaf; ML, muture leaf; SL, senescent leaf; F, flower; SE, sepal of flower in anthesis; IMG, immature green fruit; MG, mature green fruit; B, breaker fruit; B4, 4 days after breaker fruit; B7, 7 days after breaker fruit. The relative expression of *SlGLO1* in the four-whorl floral organs (**D**) of WT. Se, sepal; Pe, petal; St, stamen; Ca, carpel. Each value represents the mean ± SE of three replicates.

In order to explore the potential function of MADS-box genes playing in reproductive organ development in tomato, we isolated a tomato floral homeotic protein FBP1-like gene (namely *SlGLO1*) from wild-type tomato flowers based on a cDNA clone (GenBank accession no. XM_004245154). Nucleotide sequence analysis showed that *SlGLO1* was consistent with the cDNA clone, and contained an open reading frame (ORF) of 633 nucleotides and encoded 210 amino acid residues with an estimated molecular mass of 24.74 kDa. The search of conserved structure domains revealed that SlGLO1 protein possesses typical MADS domain, and its theoretical isoelectric point is 8.4. The sequence alignment conducted by DNAMAN 5.2.2 program showed that tomato SlGLO1 shared 90.5% identity with the *petunia* PhFBP1 (Q03488.1) protein at the amino acid level. Phylogenetic and amino acid homology analysis showed that SlGLO1 was highly homologous to PhFBP1 (syn. PhGLO1) and NbGLO1 (Fig. 1A) and belongs to a very conservative MADS-box transcription factor family (Fig. 1B).

To extend our understanding about the role of *SlGLO1* playing in tomato growth and development, we examined its expression patterns in a wide range of tomato organs, including vegetative tissues such as young, mature and senescent leaves, stems and roots, and reproductive tissues such as sepals, petals, stamens, carpels and fruits at different stages of development. Quantitative PCR technology was performed to analyze the expression of *SlGLO1*. The results showed that the expression level of *SlGLO1* was higher in flower than other tissues (Fig. 1C). Expression analysis of the four-whorl flower organs in tomato showed that the *SlGLO1* mRNA was highly accumulated in petals and stamens (Fig. 1D), which was coincidence with the study of Geuten and Irish where they show *SlGLO1* is expressed in incipient petal and stamen primordia in early developmental stages by *in situ* hybridization^9^. These results indicate that *SlGLO1* may have a specialized function in flower development process.

### Silencing of *SlGLO1* in tomato causes green petals with shorter size

**Figure 2:**
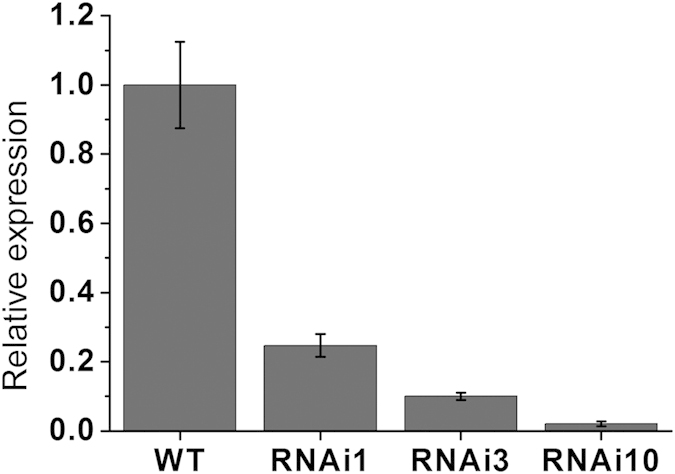
Expression profiles of *SlGLO1* between WT and *SlGLO1*-RNAi lines. The expression data of WT plants were normalized to 1. Each value represents the mean ± SE of three replicates.

To explore the physiological role of *SlGLO1* in greater depth, we obtained 10 independent *SlGLO1*-silenced lines by RNA interference (RNAi). To confirm the repression efficiency of *SlGLO1* in the transgenic lines, total RNA was extracted from flowers in WT and transgenic lines. Quantitative PCR (qPCR) results showed that *SlGLO1* transcripts were significantly reduced in 10 independent transgenic lines compared with the wild type. Among which, the accumulation of *SlGLO1* transcripts in lines 3 and 10 was remarkably reduced to roughly 2–10% of control levels, while only about 75–80% of transcript accumulation was repressed in the other lines (Fig. 2). Thus lines 3 and 10 with the strongest down regulation were selected for further investigation.

**Figure 3:**
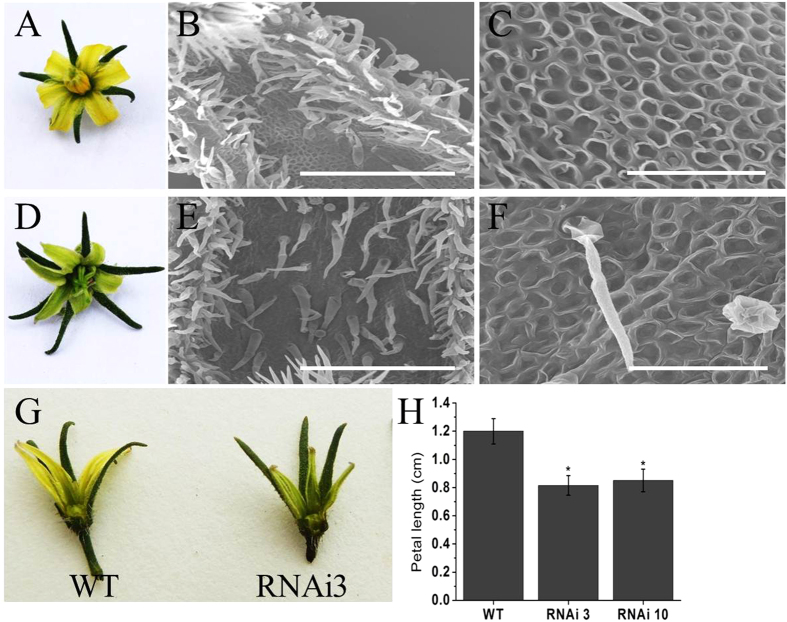
Phenotypes of petals in *SlGLO1*-RNAi lines. Wild type, (**A**–**C**); *SlGLO1*-RNAi, (**D**–**F**). Bars = 300 μm in (**B**,**E**), 60 μm in (**C**,**F**). (**G**) is digital photograph, and (**B,C,E,F**), are scanning electron micrographs. (**A**) Open wild-type flower. (**B**,**C**) Wild-type adaxial petal surface. (**D**) Flower from *SlGLO1*-RNAi line. (**E**,**F**) *SlGLO1*-RNAi line adaxial petal surface. (**G**) Silencing of *SlGLO1* results in smaller size petal. (**H**) Petal length of WT and transgenic lines. Error bars represent the standard error of the mean (n = 10). Asterisks indicate a significant difference (*P* < 0.05) between WT and transgenic lines.

Wide-type tomato flowers usually exhibit green sepals, yellow petals and staminal cones (Fig. 3A). In the second whorl, wild-type petals contain sparse trichomes on the adaxial surface (Fig. 3B). The adaxial epidermal cells of these WT second whorl organs are small and arranged compactly (Fig. 3C). In contrast with the wild type, petals of *SlGLO1*-silenced lines showed green phenotype (Fig. 3D). Scanning electron microscopy suggested that *SlGLO1*-RNAi petals developed more trichomes (Fig. 3E) and sparse cells on the adaxial compared with WT (Fig. 3F). In addition, petals of the transgenic plants were also affected in terms of length (Fig. 3G). The length of transgenic petals showed shorter size compared with WT (Fig. 3H). These results suggest that *SlGLO1*-silenced lines develop flowers showing a classic loss-of-function phenotype of B-class genes consisting of a partial transformation of the petals into sepalloid structures.

### Silencing of *SlGLO1* produces green and aberrant stamens

**Figure 4:**
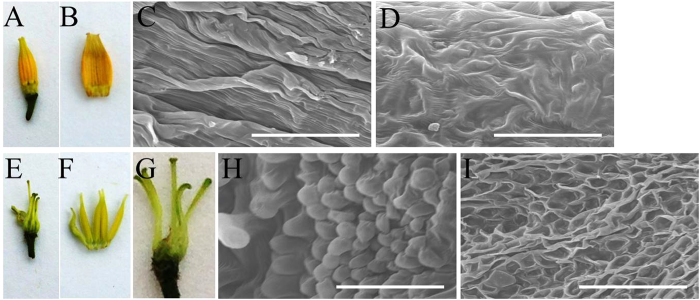
Phenotypes of stamens in *SlGLO1*-RNAi lines. Wild type, (**A**–**D**); *SlGLO1*-RNAi, (**E**–**I**). Bars = 60 μm in (**C**,**D,H**,**I)**. (**A**,**B**,**E**–**G**) are digital photographs, and (**C,D,H,I**), are scanning electron micrographs. (**A**) Wild-type stamen. **(B**) Split wild-type stamen. (**C**) Wild-type adaxial stamen proximal end. (**D**) Wild-type adaxial stamen distal end. (**E**) Stamen from *SlGLO1*-RNAi line. (**F**) Stamens of *SlGLO1*-RNAi lines are unclosed. (**G)** Silencing of *SlGLO1* results in carpelloid stamens. (**H**) *SlGLO1*-RNAi line adaxial stamen proximal end. (I) *SlGLO1*-RNAi line adaxial stamen distal end.

Wide-type tomato stamen usually exhibit yellow staminal cone in which long anthers are joined by lateral hairs (Fig. 4A,B). In the third whorl, the proximal regions of the anthers contain more elongated epidermal cells on the adaxial surface (Fig. 4C), whereas the epidermal cells are irregular in the distal regions (Fig. 4D). By contrast, *SlGLO1*-silenced stamen showed green and twisty staminal cone (Fig. 4E), suggesting that stamens are partially transformed into carpel. Besides, transgenic stamen did not close, as a result of the absence of interweaving lateral hairs in the proximal region (Fig. 4F). Occasionally, the third whorl organs, instead of forming a cone as in the wild type, were splayed out and fully transformed carpelloid stamens (Fig. 4G). Scanning electron microscopy revealed that the adaxial epidermal cells were rounded and convex in the proximal region of transgenic anthers (Fig. 4H) and concave in the distal region (Fig. 4I). These results suggest that *SlGLO1*-silenced lines develop flowers showing a typical loss-of-function phenotype of B-class genes consisting of a partial or complete transformation of the stamens into carpel-like organs. In addition, no effects were observed in sepals, pistils and vegetative organs.

### Chlorophyll content is increased and chlorophyll biosynthetic genes are significantly up-regulated in petals and stamens of *SlGLO1-*silenced lines

**Figure 5:**
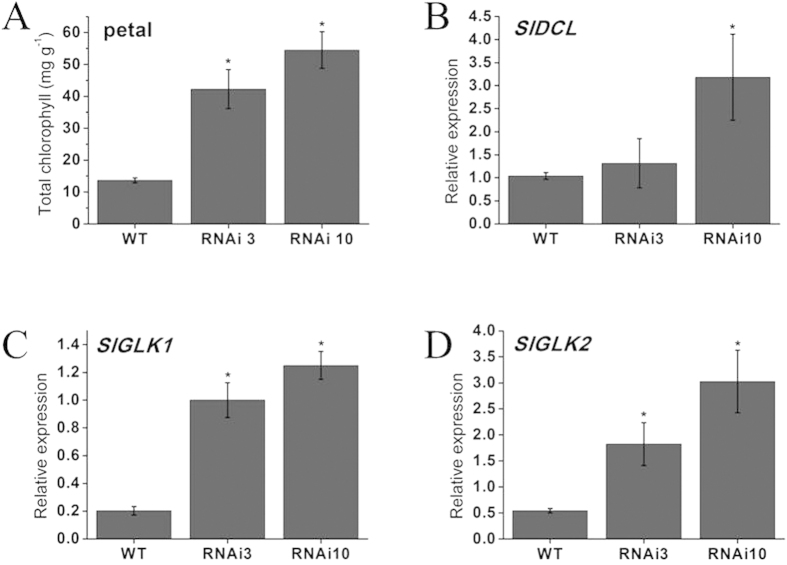
Total chlorophyll content of petals and chlorophyll biosynthetic genes expression of wild-type and silenced-*SlGLO1* lines. (**A**) Total chlorophyll content of petals from wild-type and silenced-*SlGLO1* lines. (**B**–**D**) respectively represents expression analysis of *SlDCL*, *SlGLK1*, *SlGLK2* in petals of wild-type and transgenic lines. Each value represents the mean ± SE of three replicates. Asterisks indicate a significant difference (*P* < 0.05) between WT and transgenic lines.

To determine whether the green petals phenotype represented a change in total chlorophyll content between control and *SlGLO1* RNAi lines, total chlorophyll was extracted from petals of fully opened flowers. The total chlorophyll in petal of *SlGLO1*-RNAi lines increased approximately 3- to 4-fold compared with WT (Fig. 5A), accounting for the green color of petals in transgenic lines. Furthermore, we examined the expression of positive regulators of chlorophyll biosynthetic pathway in petals of wild-type and transgenic plants, such as *DEFECTIVE CHLOROPLASTS AND LEAVES* (*SlDCL*) (GenBank: U55219), which is required for both chloroplast development and palisade cell morphogenesis in leaf mesophyll^31^, *Golden2-like1* (*SlGLK1*) (GenBank: JQ316460) and *Golden2-like2* (*SlGLK2*) (GenBank: JQ316459)^32^, which are expressed in cotyledons, sepals, and leaves. The results showed that these three chlorophyll biosynthetic genes were up-regulated significantly in petals of transgenic plants (Fig. 5B–D). Among which, *SlGLK1* and *SlGLK2* genes were up-regulated by approximate 6 folds.

**Figure 6:**
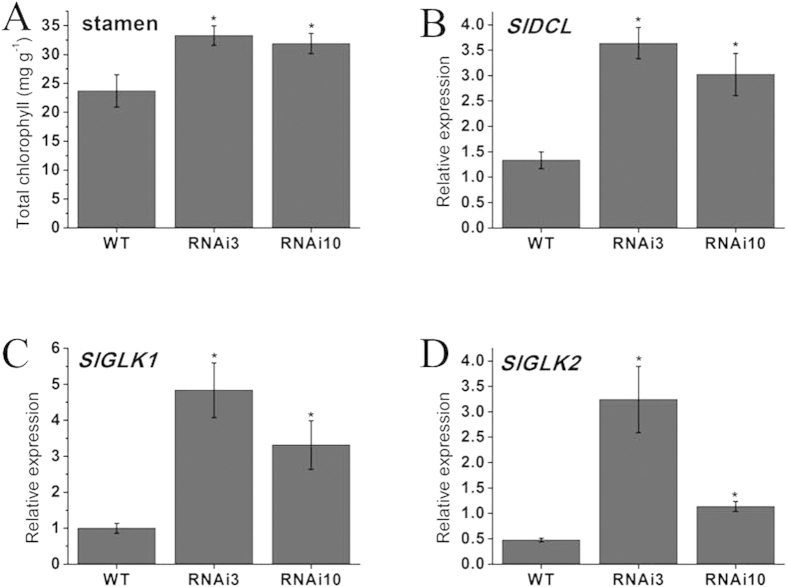
Total chlorophyll content of stamens and chlorophyll biosynthetic genes expression of wild-type and silenced-*SlGLO1* lines. (**A**) Total chlorophyll content of stamens from wild-type and silenced-*SlGLO1* lines. (**B**–**D**) respectively represents expression analysis of *SlDCL*, *SlGLK1*, *SlGLK2* in stamens of wild-type and transgenic lines. Each value represents the mean ± SE of three replicates. Asterisks indicate a significant difference (*P* < 0.05) between WT and transgenic lines.

Similarly, we assessed the total chlorophyll content in wild-type and *SlGLO1*-silenced stamens and found that total chlorophyll was also significantly increased in stamens of silenced lines (Fig. 6A). We further examined the expression of chlorophyll biosynthetic genes (*SlDCL*, *SlGLK1* and *SlGLK2*) in wild-type and *SlGLO1-*silenced stamens. The data showed that expression level of these three genes were all increased by approximately 3 to 8-fold in stamens of transgenic plants (Fig. 6B–D). These results suggest that the increased expression of chlorophyll biosynthetic genes may affect the chlorophyll biosynthesis, thus form the green petals and stamens in transgenic lines.

### Transcriptional analysis of tomato floral organ identity genes in the transgenic lines

**Figure 7:**
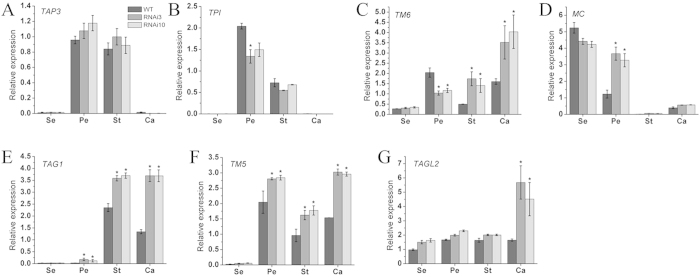
Expression Patterns of other floral organ identity genes in wild-type and RNAi Lines. Se, sepal; Pe, petal; St, stamen; Ca, carpel. (**A,B**) respectively represents expression analysis of *TAP3*, *TPI*, *TM6* (B-class genes) in wild-type and transgenic lines. (**D–G**) respectively represents expression analysis of *MC* (A-class gene), *TAG1*(C-class gene), *TM5* and *TAGL2* (E-class genes) in wild-type and transgenic lines. Each value represents the mean ± SE of three replicates. Asterisks indicate a significant difference (*P* < 0.05) between WT and transgenic lines.

Given that the *SlGLO1* gene was highly expressed at the second-whorl petal and third-whorl stamen and green petal and aberrant carpelloid stamen phenotype of *SlGLO1* RNAi plant, we examined the expression of three known tomato B-class genes (*TAP3*, *TPI*, and *TM6*) involved in the floral organ development in WT and two *SlGLO1* RNAi lines. For *TAP3* and *TPI* genes, their expression was nearly absent from first-whorl sepals but was strong in second- and third-whorl organs in wild-type floral organs. By contrast, *TM6* expression was slightly weaker in mature tomato sepals and stamens but was strongly expressed in the petals and carpels (Fig. 7A–C). In *SlGLO1* RNAi flowers, expression of *TPI* was reduced in the second-whorl organs, while *TAP3* expression was slight high in second- and third-whorl organs, expression of *TM6* was reduced in second-whorl organs and increased in the third- and fourth-whorl organs, suggesting that *SlGLO1* may regulates directly or indirectly all three other B-class genes (Fig. 7A–C).

To further characterize the potential function of *SlGLO1* in flora organ development at molecular level, a set of tomato floral homeotic genes were examined in wide type and transgenic tomato mature floral organs. *MC*, one A-class gene which is involved in sepal development^5^, was significantly up-regulated in the second-whorl petals in *SlGLO1* RNAi lines (Fig. 7D). Silencing of *SlGLO1* enhanced remarkably transcription level of *TAG1* (Fig. 7E), one C-class gene which has been identified for its role in the specification of stamen and carpel identities^16^. In addition, *TM5*^16^ and *TAGL2* (syn. *TM29*^11^,^17^), two E-class genes, were also up-regulated with various degrees in *SlGLO1* RNAi lines (Fig. 7F,G).

### *SlGLO1*-silenced tomato plants are male sterile

**Figure 8:**
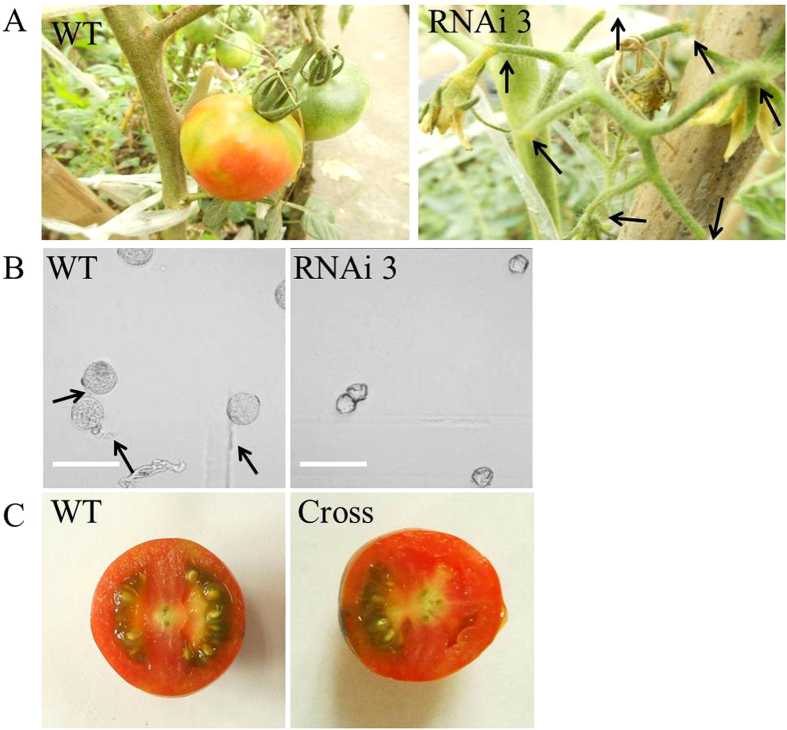
Fruit phenotype, pollen germination and cross between WT and *SlGLO1*-RNAi lines. (**A**) The *SlGLO1*-silenced lines are male sterile. (**B**) Pollen germination of WT and *SlGLO1*-RNAi lines. Pollen grains are germinated following 5 h culture at 25 °C *in vitro* containing liquid culture medium with 120 g/L sucrose, 120 mg/L boric acid, 4 mg/L gibberellin and 0.5 mg/L thiamine. Bars = 20 μm. (**C**) Fruit of the transgenic line produces normally seeds through manual crossing assay.

The development and function of the pollen is crucial important in the reproductive process of most plant species. In our study, *SlGLO1*-silenced tomato lines cannot produce fruit set (Fig. 8A). To examine whether or not the pollen collected from transgenic lines germinated, a pollen germination experiment was performed between WT and transgenic lines. The results showed that most of pollen grains in these transgenic lines were aberrant and uninflated, and failed to germinate (Fig. 8B), suggesting pollen development is markedly affected by *SlGLO1* silencing. Transgenic flowers which displayed transformation of stamens to carpelloid tissue were male sterile, but seeds developed normally when such flowers were manually crossed with wild-type pollen (Fig. 8C). This observation indicates that the ovules of such transgenic plants are functional.

**Figure 9:**
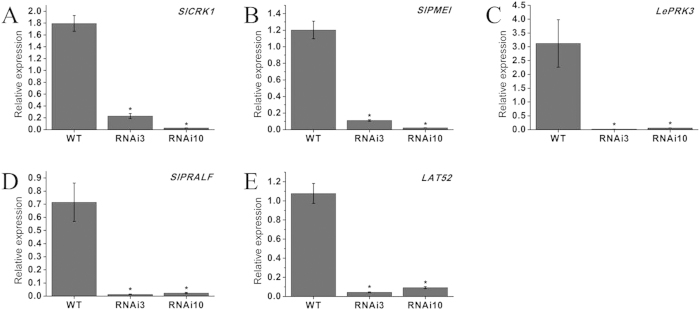
Expression analysis of tomato pollen-specific genes in the pollen of wild-type and transgenic plants. (**A–E**) respectively represents expression of pollen-specific genes *SlCRK1*, *SlPMEI*, *LePRK3*, *SlPRALF* and *LAT52* in pollen of wild-type and transgenic lines. Each value represents the mean ± SE of three replicates. Asterisks indicate a significant difference (*P* < 0.05) between WT and transgenic lines.

Further, we assessed the expression of tomato pollen-specific genes involved in the pollen development in WT and *SlGLO1*-silenced lines. *SlCRK1* is one of the cysteine-rich receptor-like kinases, is important in pathogen defense and programmed cell death^33^; Pectin methylesterase inhibitor (SlPMEI) is key regulators of pectin methylesterase (PME)^34^; Pollen-specific receptor kinases gene *LePRK3* is likely to be involved in perceiving extracellular cues during pollen tube growth^35^; Exogenous rapid alkalinization factor (SlPRALF) acts as a negative regulator of pollen tube elongation within a specific developmental window^36^ and *LAT52* may play a role during germination or early tube growth^37^. The quantitative PCR results showed that these genes were all down-regulated by more than 90% in *SlGLO1*-silenced lines (Fig. 9), which suggest that silencing of *SlGLO1* represses the expression of these genes, subsequently leads to pollen sterility.

### Analysis of putative cis-acting elements within the *SlGLO1* promoter

To date, many cis-elements have been reported for their critical roles in determining the tissue-specific expression profiles of plant genes^38^,^39^. In this study, a 1534-bp region upstream of the *SlGLO1* start codon was analyzed to identify cis-acting elements, using the information in two public databases ( http://www.dna.affrc.go.jp/PLACE and http://intra.psb.ugent.be:8080/Plant CARE). Statistical analysis revealed that there were nine pollen-specific activation-related elements POLLEN1LELAT52 (*Lat52*, AGAAA)^40^ and eight late pollen gene g10-related elements (*G10*, GTGA)^41^ enriched in the promoter of *SlGLO1* gene, which may be crucial for pollen development (Fig. S1).

## Discussion

To date, it has been demonstrated that five classes of MADS-box genes (A, B, C, D and E) determine the identities of floral organ^1^,^42^. In the ABCDE model, petal and stamen structures are specified by the genes of B-class. The B-class floral homeotic genes has been reported to be implicated in flora organs development processes in many model plants, such as, *Arabidopsis*^43^,^44^, *Antirrhinum*^45^,^46^, *Petunia*^26^,^47^, *Nicotiana*^9^ and tomato^8^,^9^,^10^. For instance, the flowers of transgenic *petunia* plants in which *PhFBP1* (syn. *PhGLO1*) expression is inhibited by a co-suppression approach, exhibit homeotic conversions of petals towards sepals and stamens towards carpels^47^. In *petunia*, loss of function of *GREENPETALS* (*GP*, syn. *PhDEF*) affects petal identity, whereas *TM6* is required for stamen identity^26^,^48^. In *Nicotiana*, *DEF*-silenced plants display a marked transformation of petals into sepals and stamens into carpeloid structures^49^. Most *Nicotiana* flowers of *NbGLO1*-VIGS plants develop sepaloid tissues in petals and carpelloid tissues in stamens^9^. Here, we characterized the function of tomato *SlGLO1* by RNAi-mediated gene silencing. Similar to the reports in *Nicotiana* and *Petunia*^9^,^47^, the silencing of *SlGLO1* in tomato causes floral organ abnormal phenotypes, including green petals and stamens, shorter petals size, aberrant carpelloid stamens and defective pollen grains (Figs 3, 4 and 8), suggesting *SlGLO1* plays an important role in diverse developmental processes.

RNAi repression of *SlGLO1* resulted in green petals phenotype (Fig. 3D). Similarly, repression of *PhGLO1* (syn. *PhFBP1*) in *Petunia* results in green tips at the end of the midveins of the petals^47^. Similar alteration of petals coloration phenotype is also observed in *Petunia phglo1* mutant: the midveins of the petals become broader and greener, especially toward the edge of the corolla at the abaxial side of the petals^24^. Furthermore, Comparing the total chlorophyll contents of transgenic petals with wild type mature flowers, total chlorophyll contents in transgenic lines were all significantly increased, while that in wild-type remain at low level (Fig. 5A). Expression levels of chlorophyll biosynthetic genes *SlDCL*, *SlGLK1*, and *SlGLK2*, were increased in petals of transgenic plants compared with wild-type (Fig. 5B–D). These results suggest that the silencing of *SlGLO1* leads to the activation of the chlorophyll biosynthesis, and then results in green petals. Thence, we speculate that *SlGLO1* may be involved in the activation of chlorophyll synthesis. Besides, the *SlGLO1*-silenced lines represented shorter petals size (Fig. 3G,H). A decrease in cell proliferation (Fig. 3F) in the *SlGLO1*-RNAi plants may partially explain the phenotype of the smaller petals size. Similar phenotype is observed in *Petunia PhGLO1* (syn. *PhFBP1*) transgenic plants^47^, tomato *TM6* RNAi Line^10^, and *Nicotiana NbGLO1*-VIGS plants^9^. However, previous studies showed that in tomato cv. Micro-Tom (a dwarf cultivar of tomato), silencing of *SlGLO1* has no obvious effect on petal development^9^. The tomato cultivar Micro-Tom was produced for ornamental purposes by crossing Florida Basket and Ohio 4013-3 cultivars, and displays a very dwarf phenotype with small and red ripened fruits. Based on its pedigree, the phenotype of Micro-Tom plants is due to at least three mutations: *self-pruning* (*sp*), *dwarf* (*d*) and *miniature* (*putative mnt*)^29^,^30^. The characterization of one of them, *putative mnt*, has still been not clarified. Recent study indicated that Micro-Tom seedlings in the dark present a weak photomorphogenic phenotype (disappearance of hook and cotyledon opening)^50^. We speculate that mutant tomato Micro-Tom results in a weak photomorphogenesis and thus some genes participating in light signal transduction pathways may be mutated. In our study, we used tomato cv. Ailsa Craig (a near-isogenic tomato line), as the research material which is normal height compared with Micro-Tom. This may explain the difference in phenotype observed as compared to the study previously mentioned.

In our study, *SlGLO1* also impacts stamens development. The *SlGLO1*-silenced lines produced green stamens (Fig. 4E). Total chlorophyll content in transgenic lines was significantly increased, while that in wild-type accumulated little (Fig. 6A). Expression levels of chlorophyll biosynthetic regulatory genes including *SlDCL*, *SlGLK1*, and *SlGLK2*, were increased in stamens of transgenic plants compared with wild-type (Fig. 6B–D). Besides, transgenic stamen showed an unclosed phenotype (Fig. 4F). However, these phenotypes were not found in previous studies in *Micro-Tom* by Geuten and Irish^9^. Aberrant carpelloid stamens were also observed in transgenic lines (Fig. 4E,G). These results suggest that *SlGLO1*-silenced lines develop flowers showing a classic B-class gene loss-of-function phenotype consisting of a partial or complete transformation of the stamens into carpel-like organs.

Whether silencing of *SlGLO1* affects other floral organ identity genes? We investigated the expression of these genes in WT and transgenic flowers. We performed a quantitative analysis of the transcripts of *TM6* (syn. *TDR6*^11^,^12^), *TPI*^10^ (syn. *SlGLO2*^7^) and *TAP3* gene (syn. *SlDEF*, *LeAP3*^10^,^13^) in the WT and *SlGLO1* RNAi lines (Fig. 7A–C). The results found that repressed *SlGLO1* increased expression of *TAP3* and reduced the expression of *TPI* in the second- and third-whorl organs of *SlGLO1*-RNAi plants, which was consistent with the study of Geuten and Irish^9^. In the study of Geuten and Irish, knockdown of *SlGLO1* results in reduced second-whorl *TPI/SlGLO2* expression, and knockdown of *TPI/SlGLO2* causes a reduction in *SlGLO1* expression, suggesting cross-activation of *TPI/SlGLO2* and *SlGLO1* in the second-whorl organs. Besides, silencing of *SlGLO1* down-regulated the expression of *TM6* in the second-whorl organs of *SlGLO1*-RNAi plants. Therefore, these results suggest that *SlGLO1* may regulate all three other B-class genes directly or indirectly. According to our results, silencing of *SlGLO1* modified the expression profiles of *TAP3*, *TPI* and *TM6* in flowers, indicating that the transgenic flower phenotype may be due to a coordinated misregulation of *TAP3*, *TPI*, and *TM6*. Furthermore, to further characterize the potential molecular regulation mechanism of *SlGLO1* in flora organ development, the other floral organ identity were tested in mature floral organs of wide type and transgenic tomato (Fig. 7D–G). Our results showed that silencing of *SlGLO1* affected significantly the expression of A-class (*MC*), C-class (*TAG1*) and E-class (*TM5* and *TAGL2*) genes. Silencing of *SlGLO1* increased the transcript levels of *MC*, *TAG1*, *TM5* and *TAGL2* significantly, suggesting that *SlGLO1* may be involved in the repression of these genes. Together, these results indicate that the *SlGLO1* in tomato affects floral organ development possibly via regulating the expression of the other floral organ identity genes.

B-class MADS-box genes are also involved in the pollen development. For instance, specific *PFGLO2* or/and *PFTM6* silencing show a reduction in mature pollen in *Physalis floridana*^25^. In our study, we performed a pollen germination experiment between WT and transgenic lines. The result showed that most of pollen grains in these transgenic lines were aberrant and failed to germinate (Fig. 8B), suggesting silencing of *SlGLO1* affects markedly pollen grains development and forms defective pollen grains. In addition, *SlGLO1*-silenced stamens were twisty and seem to become shorter, relatively. These alterations in stamens and pollen grains were susceptible for pollination. Therefore, the *SlGLO1*-silenced lines were male sterile. However, it is demonstrated that the ovules of transgenic lines were functional through manual crossing assay (Fig. 8C). Previous studies about two pollen-specific genes including *SlCRK1* and *SlPMEI* showed that their promoters region possess lots of pollen-specific cis-acting elements, such as, the pollen-specific activation-related elements POLLEN1LELAT52 (Lat52, AGAAA) and late pollen gene g10-related elements (G10, GTGA)^33^,^34^. Kim *et al.*^33^,^34^ also confirmed promoters of *SlCRK1* and *SlPMEI* have strong pollen-specific activity in the homozygous transgenic plants of tomato. Similarly, we analyzed putative cis-acting elements within the *SlGLO1* promoter. The result showed that there were nine pollen-specific activation-related elements POLLEN1LELAT52 (*Lat52*, AGAAA) and eight late pollen gene g10-related elements (*G10*, GTGA) in the promoter of *SlGLO1* gene (Fig. S1). This result demonstrates further that *SlGLO1* may be involved in the pollen development. Furthermore, expression analysis of tomato pollen-specific genes in transgenic lines suggest that *SlGLO1* may regulates directly or indirectly all these pollen-specific genes through combining with these genes promoters or interacting with other transcription factors.

In conclusion, this study concerned the morphological, physiological and molecular features of *SlGLO1*-RNAi transgenic tomato. Though a large amount of study focused on B-class genes, the specific biological functions of most plant B-class genes still remain to be elucidated. Clearly, understanding the role of *SlGLO1* gene will not only extend knowledge of the biological function of B-class genes, but also provide new insight into exploring further the significance of *SlGLO1* in regulating plant floral organ development.

## Materials and Methods

### Plant materials and growth conditions

In our study, we used tomato (*Solanum lycopersicon* Mill. cv. Ailsa Craig), a near-isogenic tomato line, as the wild type. The plants were grown in a greenhouse and managed routinely. Transgenic cultures grew under standard greenhouse conditions (16-h-day/8-h-night cycle, 25 °C/18 °C day/night temperature, 80% humidity, and 250 μmol m^−2^ s^−1^ light intensity). Tomato plants of the first generation (T0) came from tissue culture were used in our study. For organ-specific expression profiling of *SlGLO1*, the tissues of roots, stems, leaves, sepals, flowers, and fruits of different period from tomato plant were collected. The ripening stages of tomato fruits were divided following the method described by Xie *et al.*^6^. The four-whorl mature floral organs (sepal, petal, stamen and carpel) in the wild type and silenced lines were also collected. All plant samples were immediately frozen with liquid nitrogen, mixed, and stored at –80 °C until further use.

### Isolation and sequence analysis of *SlGLO1*

The primers: Forward, 5′ AAATAGGGGAAGTGTTTGAGC 3′ and Reverse, 5′ GAACCAAACCACATCACAAGA 3′ were used for cloning the coding region of *SlGLO1*. The PCR procedure was performed at 94 °C for 5 min followed by 35 cycles of 30 s at 94 °C, 30 s at 56 °C and 30 s at 72 °C, and a final extension at 72 °C for 10 min. The amplified products were subcloned into pMD18-T vector (Takara, Dalian, China), and sequenced. The translation of *SlGLO1* gene was used by the ExPASy translate tool, while grand average of hydropathicity (GRAVY) and theoretical molecular weight (Mw) were calculated using the ExPASy ProtParam tool ( http://www.expasy.org/). Sequence alignment was conducted using the DNAMAN 5.2.2 program. Conserved structure domains were annotated according to ScanProsite ( /scanprosite/). An unrooted neighbor-joining tree was constructed using the MEGA 5.05 software. To analyze putative cis-elements in the promoter region of *SlGLO1* gene, promoter sequence (1500 bp regions upstream the 5′ end of the predicted ORF) of *SlGLO1* gene was extracted from SGN database and searched against the promoter database PLACE ( http://www. Dna. Affrc. Go. jp/PLACE/signalscan.html)^51^ and plant CARE ( http://bioinformatics. Psb.ugent. be/webtools/plantcare/html/).

### Construction of the *SlGLO1* RNAi vector and plant transformation

In order to repress the expression of the *SlGLO1* gene, an RNAi vector was constructed. A 395-bp specific DNA fragment of *SlGLO1* was amplified from wild-type flower cDNA with the following primers: Forward, 5′-GGTACCAAGCTTAAATAGGGGAAGTGTTTGAGC-3′ and Reverse, 5′-CTCGAGTCTAGAGAACCAAACCACATCACAAGA-3′, which had been tailed with KpnI/*Hin*dIII and *Xho*I/*Xba*I restriction sites at the 5′ end, respectively. Then, the amplified products were digested with *Hin*dIII/*Xba*I and *Kpn*I/*Xho*I and inserted into the pHANNIBAL plasmid at the *Hin*dIII/*Xba*I restriction site in the sense orientation and at the *Kpn*I/*Xho*I restriction site in the antisense orientation. Finally, the double-stranded RNA expression unit, containing the cauliflower mosaic virus (CaMV) 35 S promoter, *SlGLO1* fragment in the antisense orientation, PDK intron, *SlGLO1* fragment in the sense orientation, and OCS terminator, was purified and linked into the plant binary vector pBIN19 with *Sac*I and *Xba*I restriction sites.

The generated binary plasmids were transformed into WT tomato cotyledon explants via *Agrobacterium strain* LBA440469^52^. Positive transgenic lines were screened for kanamycin (50 mg L^−1^) resistance. The transgenic plants were detected with primers NPTII-F (5′-GACAATCGGCTGCTCTGA-3′) and NPTII-R (5′-AACTCCAGCATGAGATCC-3′). The positive transgenic plants were selected and used for subsequent experiments.

### Quantitative real-time PCR analysis

Total RNA was isolated using Trizol (Invitrogen, Shanghai, China) according to the manufacturer’s instructions and was treated with the Dnase-I (Promega, Beijing, China) for 30 min at 25 °C. The first strand cDNA synthesis was performed using M-MLV reverse transcriptase (Promega, Beijing, China) with oligo (dT)_18_ primer. Quantitative RT-PCR was carried out using the CFX96TM Real-Time System (Bio-Rad, USA). All reactions were performed using the SYBR Premix Ex Taq II kit (Takara) in a 10 μL total sample volume (5.0 μL of 2 × SYBR Premix Ex Taq, 1.0 μL of primers, 1.0 μL of cDNA, and 3 μL of distilled, deionized water). Quantitative RT-PCR reactions were performed using a two-step method: 95 °C for 30 s, followed by 40 cycles of 95 °C for 5 s, and 60 °C for 30 s. To remove the effect of genomic DNA and the template from the environment, no-template control and no-reverse transcription control experiments were performed. Tomato *CAC* gene was selected as internal standard for organ-specific expression analysis^53^. The primers *SlGLO1*-Q-F and *SlGLO1*-Q-R (Table S1) were used to determine the expression levels of *SlGLO1* in the wild type and transgenic lines. The analysis of relative gene expression levels were detected using the 2^−ΔΔС^_T_ method^54^. Additionally, all samples were taken in three biological replicates and standard curves were run simultaneously. Primers used for quantitative RT-PCR are showed in Supplementary Table S1.

### Extraction and quantitation of petal and stamen chlorophyll

Weighted 0.5 g fresh petals of wild-type and transgenic lines, pounded to powder with liquid nitrogen, extracted with 5 ml 80% acetone for 24 h in dark, centrifuged 5000 rpm for 15 min at 4 °C. The absorbance of the supernatant was measured at 645 and 663 nm in a PerkinElmer Lambda 900 UV/VIS/NIR spectrophotometer using above-mentioned 80% acetone as a blank. Total chlorophyll content were calculated using the formulas: Chl (mg g^−1^) = 20.29A645 + 8.02A663^55^. The chlorophyll of each sample was extracted and measured in triplicate. Chlorophyll contents of stamens were measured using the same method.

### Scanning electron microscopy

Petals and stamens of wild type and transgenic plants at anthesis were fixed in 70% ethanol/acetic acid/formaldehyde (18:1:1, by volume; FAA), and subsequently dehydrated in an ethanol-water series. After dissection, the materials were dried and sputtered gold for scanning electron microscopy according to a previously published protocol^56^.

### Statistics of petals length

In our study, we found that transgenic petals were shorter than the wild type. We measured the length of petals at the fully open flowers, at least 10 flowers per plant were measured.

### Pollen collection, handling and germination

Pollen was collected in early July from mature floral organs of tomato plants cultivated in the glasshouses. Flowers were collected during the early morning and pollen was extracted within 1–2 h of collection as described by Karapanos *et al.*^57^. The pollen was then enclosed *in vitro* containing silica gel and stored for up to one week at 4 °C. Liquid culture medium for tomato pollen germination was composed of 120 g/L sucrose, 120 mg/L boric acid, 4 mg/L gibberellin and 0.5 mg/L thiamine^58^. After 5 hour incubation at 25 ± 1 °C in dark condition, 2 μl of pollen culture solution were placed on microscope slides and photographs were taken at 4 sites per slide, with the aid of an optical microscope (Olympus BX 40, Olympus Corp., Tokyo, Japan). A pollen grain was considered germinated when the pollen tube was equal or larger than the grain diameter (25–30 μm)^59^,^60^. The experiments were repeated three times.

### Cross assay

Unopened flower buds (about 1 cm in length) from transgenic lines were sliced open lengthwise and emasculated with forceps. Mature pollen from the wild type tomato was transferred by brushing anthers onto the stigmas of transgenic lines. Pollinated flowers were labeled and bagged with small plastic bags to prevent uncontrolled cross-pollination.

### Statistical analysis

The mean values of data were measured from three replicates and ‘Standard Error’ of the means was calculated. Data were analyzed by Origin 8.0 software, and *t* test (SAS 9.2) was used for assessing significant differences among the means.

## Additional Information

**How to cite this article**: Guo, X. *et al.* The tomato floral homeotic protein FBP1-like gene, *SlGLO1*, plays key roles in petal and stamen development. *Sci. Rep.* **6**, 20454; doi: 10.1038/srep20454 (2016).

## Supplementary Material

Supplementary Information
