# Supplementary material for: The tomato floral homeotic protein FBP1-like gene, SlGLO1, plays key roles in petal and stamen development
Source: Sci Rep. 2016 Feb 4;6:20454. doi: 10.1038/srep20454 (PMC4740859; doi:10.1038/srep20454)
Supplement: Supplementary Information [file srep20454-s1.pdf]

Title:

The tomato floral homeotic protein FBP1-like gene, *SlGLO1*, plays key roles in petal and stamen development

Authors:

Xuhu Guo, Zongli Hu, Wencheng Yin, Xiaohui Yu, Zhiguo Zhu, Jianling Zhang, Guoping Chen*

Key Laboratory of Biorheological Science and Technology (Chongqing University), Ministry of Education, Bioengineering College, Chongqing University, Chongqing 400044, People's Republic of China

Table S1. Primers used for Quantitative PCR analysis

Primer code	Primer sequences (5' → 3')	Accession number	Application
CAC-Q-F	CCTCCGTTGTGATGTAACTGG	SGN-U314153	Internal standard gene for Quantitative RT-PCR in tomato development
CAC-Q-R	ATTGGTGGAAAGTAACATCATCG		
SlGLO1-Q-F	GCTTACTGGAAGAAGATTGTGGG	XM_004245154	Quantitative RT-PCR analysis for *SlFBP1*
SlGLO1-Q-R	CTCATTCTGTTTTTCACGGATACC		
SlDCL-Q-F	CCGCAAGGATGGTGAAACA	U55219	Quantitative RT-PCR analysis for chlorophyll biosynthetic genes
SlDCL-Q-R	TCCGCTTCCGAAAATGCC		
SlGLK1-Q-F	GAATTTTCCGTAAGCAGTGGTG	JQ316460	
SlGLK1-Q-R	CTTCTCCTTGATTTAGGCTCGT		
SlGLK2-Q-F	ACAATCGGAGGCGGAGGA	JQ316459	
SlGLK2-Q-R	CAAGGAGTGCCTGGTACAAGAG		
MC-Q-F	AAGTAGCAGAAGCAAGGAGGA	AF448521	Quantitative RT-PCR analysis for floral organ identity genes
MC-Q-R	CAAGCGATTAGCAAAGAGTGA		
TAP3-Q-F	TATAAGTCCCTCAATCACGACCA	DQ674532	
TAP3-Q-R	GATCATTTAGGCTTTCTCCCATC		
TPI-Q-F	TCTGGGAGGAGACTATGGGATG	DQ674531	
TPI-Q-R	TCAGACTGCTTGGCACTGATACTA		
TM6-Q-F	CTACAACCATTGCACCCCAAT	AY098734	
TM6-Q-R	CAGGAGAGACGTAGATCACGAGAA		
TAG1-Q-F	ATGAACTTGATGCCAGGGAGT	AY098733	
TAG1-Q-R	GGGGTTGGTCTTGTCTAGGGTA		
TM5-Q-F	CTTTGTGATGCTGAGGTTGCTC	X60758	
TM5-Q-R	TTTCCAGTGCTTCTCGTGTTG		
TAGL2-Q-F	CAGCAGCAACATCCTCAATCTC	AY098738	
TAGL2-Q-R	CACAGCATCCAACCAGGTATCA		
SlCRK1-Q-F	AAAGGGATTCTTCCTGATGGC	KC736926	Quantitative RT-PCR analysis for floral organ identity genes
SlCRK1-Q-R	TCTCGGGTCCTTCTATGCTACA		
SlPMEI-Q-F	AAACTCCTATCATTCCAAAACCC	KC736927	
SlPMEI-Q-R	CAATTGCATCTTCATACACCTCTT		
LePRK3-Q-F	TGTCTGTCGTGGTGAAGAGGTT	AF243040	
LePRK3-Q-R	AGCTGAGCATGTGCTGTCCC		

SlPRALF-Q-F	CTTCCTTCTTCAACGACCCTG	SGN-U324197
SlPRALF-Q-R	CATCGCCCTGTAACTAATGTGG	
LAT52-Q-F	TAATGGAGACCACGAGAACGA	X15855
LAT52-Q-R	GGGAATAAACCCAACTCATCAAG	

Figure S1 | Schematic representation of the *SlGLO1* promoter. The putative cis-regulatory promoter sequences are underlined. The locations of pollen-specific cis-elements were identified using the PLACE and plant CARE databases and the symbols of each cis-element are annotated below.

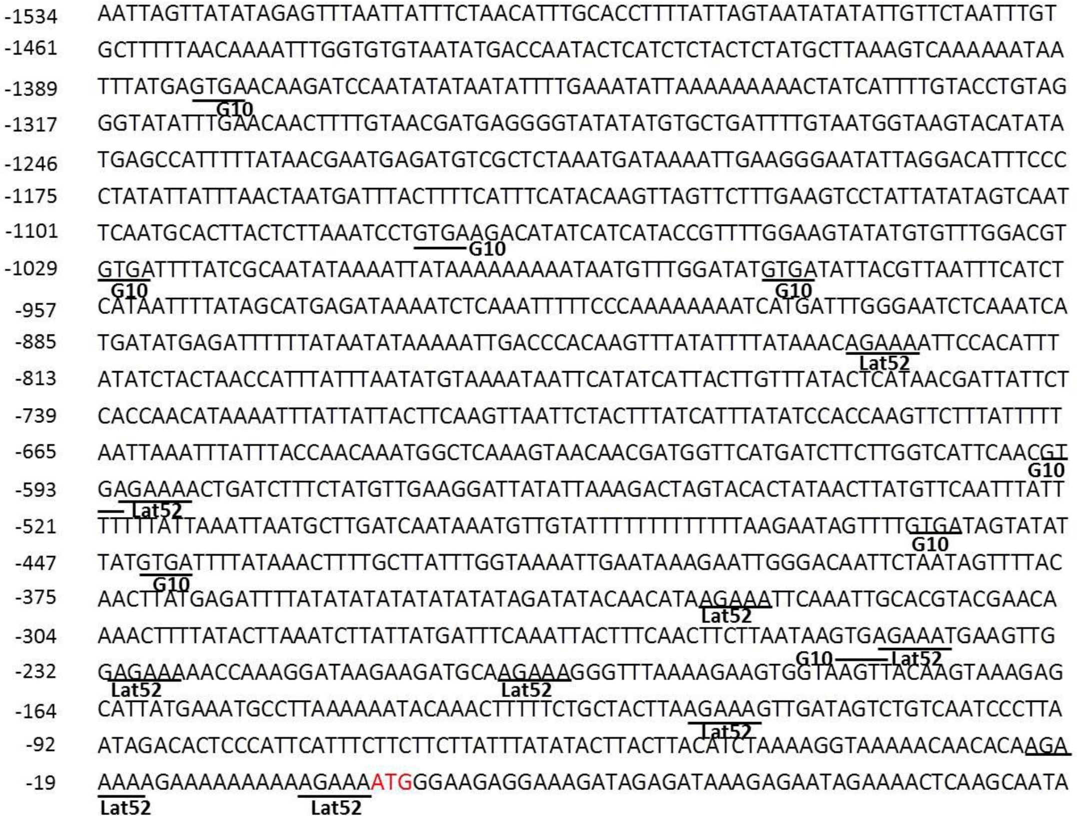

Lat52: Pollen specific activation

G10: Homologue of the tomato gene *lat56*
